# Neurotoxicity in Snakebite—The Limits of Our Knowledge

**DOI:** 10.1371/journal.pntd.0002302

**Published:** 2013-10-10

**Authors:** Udaya K. Ranawaka, David G. Lalloo, H. Janaka de Silva

**Affiliations:** 1 Faculty of Medicine, University of Kelaniya, Ragama, Sri Lanka; 2 Liverpool School of Tropical Medicine, Liverpool, United Kingdom; Women's and Children's Hospital, North Adelaide, Australia

## Abstract

Snakebite is classified by the WHO as a neglected tropical disease. Envenoming is a significant public health problem in tropical and subtropical regions. Neurotoxicity is a key feature of some envenomings, and there are many unanswered questions regarding this manifestation. Acute neuromuscular weakness with respiratory involvement is the most clinically important neurotoxic effect. Data is limited on the many other acute neurotoxic manifestations, and especially delayed neurotoxicity. Symptom evolution and recovery, patterns of weakness, respiratory involvement, and response to antivenom and acetyl cholinesterase inhibitors are variable, and seem to depend on the snake species, type of neurotoxicity, and geographical variations. Recent data have challenged the traditional concepts of neurotoxicity in snake envenoming, and highlight the rich diversity of snake neurotoxins. A uniform system of classification of the pattern of neuromuscular weakness and models for predicting type of toxicity and development of respiratory weakness are still lacking, and would greatly aid clinical decision making and future research. This review attempts to update the reader on the current state of knowledge regarding this important issue.

## Introduction

Snakebite is a neglected tropical disease of global importance [1]. Kasturiratne et al. (2008) estimated that annually at least 1.2 million snakebites, 421,000 envenomings, and 20,000 deaths occur due to snakebite worldwide [2]. The actual figures are likely to be much higher than these estimates. A study in a rural Sri Lankan community found that nearly two-thirds of snakebite related deaths are not reported in hospital-based data [3]. A nationally representative survey in Bangladesh suggested that incidence of snakebite is much higher than previously estimated [4]. Data from the Million Deaths Study in India estimates that snakebite deaths are more than 30-fold higher than recorded in official hospital returns [5].

Snakebite-related mortality is highest in resource-poor countries, and is directly related to socioeconomic indicators of poverty [1]. The highest burden of morbidity and mortality related to snakebite is seen in the rural poor communities of tropical countries in South Asia, Southeast Asia, and sub-Saharan Africa [2], [6], [7]. Increased exposure to snakes due to traditional agricultural practices, lack of good health care services, poor access to available services, influence of health-seeking behaviour on accessing the available health care services, and lack of effective antivenom all contribute to this [2], [8].

Neurotoxicity is a well-known feature of envenoming due to elapids (family Elapidae) such as kraits (*Bungarus* spp.) [9]–[28], cobras (*Naja* spp.) [9], [14], [20], [21], [29]–[39], taipans (*Oxyuranus* spp.) [40]–[46], coral snakes (*Micrurus* spp.) [47]–[51], death adders (*Acanthophis* spp.) [52]–[54], and tiger snakes (*Notechis* spp.) [55]–[57]. It has also been well described with pit vipers (family Viperidae, subfamily Crotalinae) such as rattlesnakes (*Crotalus* spp.) [58]–[67]. Although considered relatively less common with true vipers (family Viperidae, subfamily Viperinae), neurotoxicity is well recognized in envenoming with Russell's viper (*Daboia russelii*) in Sri Lanka and South India [9], [68]–[75], the asp viper (*Vipera aspis*) [76]–[82], the adder (*Vipera berus*) [83]–[85], and the nose-horned viper (*Vipera ammodytes*) [86], [87].

Acute neuromuscular paralysis is the main type of neurotoxicity and is an important cause of morbidity and mortality related to snakebite. Mechanical ventilation, intensive care, antivenom treatment, other ancillary care, and prolonged hospital stays all contribute to a significant cost of provision of care. And ironically, snakebite is common in resource-poor countries that can ill afford such treatment costs. The cost of neurotoxic envenomation is easily overlooked in the face of high mortality, and surprisingly, there are few data on the cost of caring for patients with neurotoxic envenomation.

Several other acute neurological features are reported after snake envenomation, which are likely to be due to direct neurotoxicity. These have not been well studied, with available data being mostly confined to case reports, and their potential pathophysiological mechanisms remain unclear. Neurological manifestations can also result from non-neurotoxic effects of envenoming, such as cerebral haemorrhage and infarction due to coagulopathy, and myotoxicity. This article will focus only on the direct neurotoxic effects of envenoming.

There are many challenges to the study of neurotoxicity after snakebite. There is considerable variation between individual patients in the clinical manifestations following envenoming by any particular species. Clinical presentations of neurotoxicity are likely to be colored by the emotional response to a snakebite, neurological changes related to hypotension, shock and other organ dysfunction (such as renal impairment), and by the non-neurotoxic neurological manifestations of envenoming such as those due to coagulopathy. Comparing findings from different studies is difficult, as there is a lack of uniformity in description or grading of neuromuscular weakness, or in assessment of response to treatment. Interpretation of neurophysiological findings is also difficult as different methodologies have been used between studies.

The effects of a bite from one snake species can also vary, as venom constituents in one species may vary seasonally, geographically, as well as ontogenetically, and some venoms contain a number of different neurotoxins.

Accurate case definition is the key to meaningful interpretation of available data and comparison between studies. However, this is hampered by the difficulties in identifying envenoming snakes, which have been previously highlighted [88]–[91]. Previous studies on snake identification have yielded variable results [92], [93]. Immunodiagnosis of snake venom antigen is the most reliable way of identifying the biting species, but cost and availability issues preclude its use in the resource-poor settings where snakebite is common [88]–[91]. Identification of the killed snake by trained health staff is perhaps the best alternate way, but snake identification without specific training can be incorrect and can potentially lead to serious mistakes in management. Also, rates of snake capture are low in most series, varying from 5–30% [14], [94]–[96]. Use of a clinical scoring system [89] or a syndromic classification [90] has been suggested to improve snake identification in the community setting, but these approaches have their own drawbacks.

## Methodology

We searched PubMed with varying combinations of the search terms “snake bite,” “snake envenoming,” “snake venom,” “neurotoxicity,” “neurology,” and “neurological manifestations.” We acquired only English-language articles. We also obtained articles on “neuromuscular junction” and “neuromuscular block.” Additional related articles were obtained from citation tracking of retrieved articles and tracking of “related citations” in PubMed. Altogether, 624 titles and abstracts were screened, and 287 full articles were retrieved and read by a single author (UKR) for data acquisition. The reference list was further modified following reviewers' comments.

## Pathophysiological Basis of Neuromuscular Paralysis

**Figure 1:**
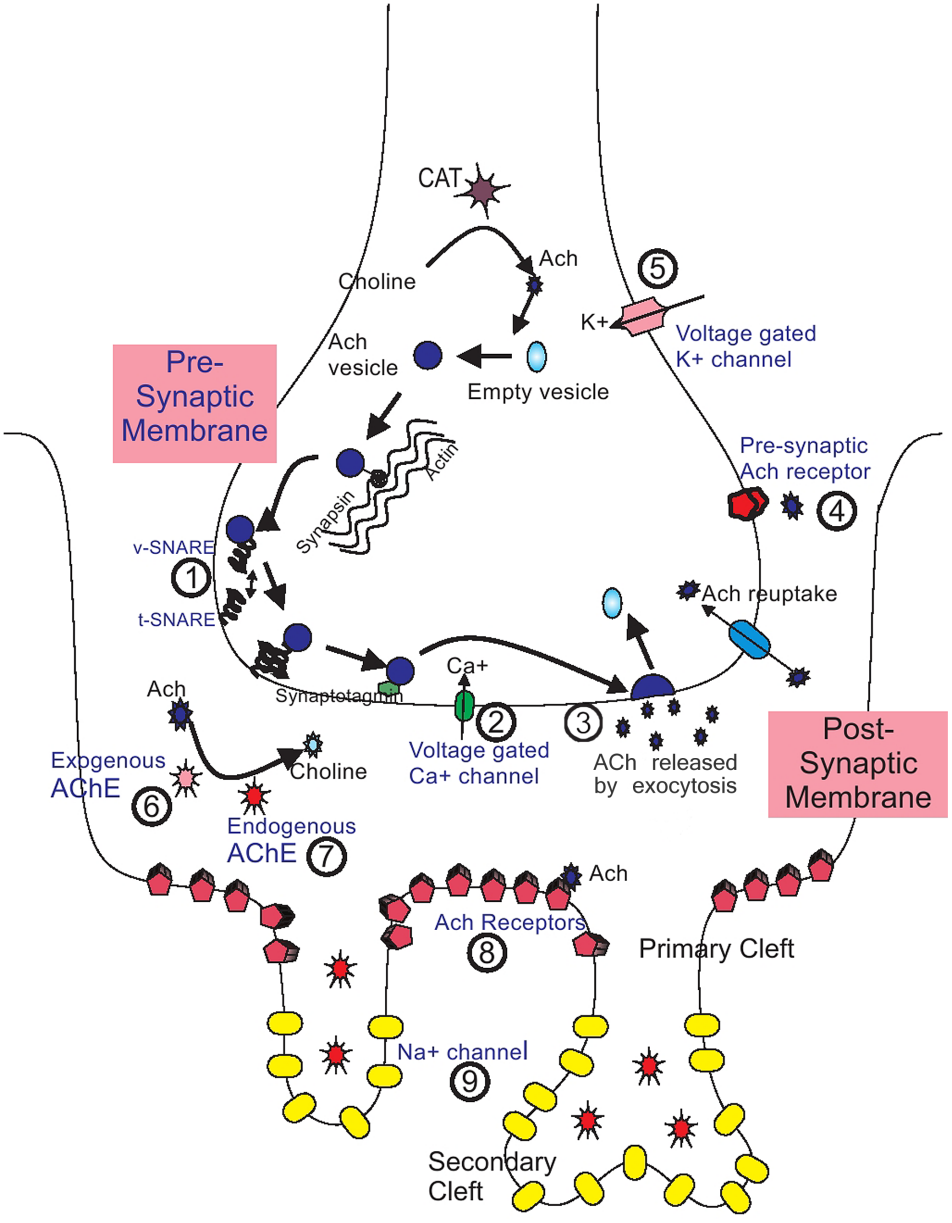
Sites of action of snake neurotoxins and other substances on the neuromuscular junction. Schematic representation of the neuromuscular junction showing different sites of action of snake neurotoxins, other toxins, and pharmacological substances, and sites of involvement in disease states (examples indicated where relevant). 1. **Synaptic vesicular proteins**: *Snake toxins*: beta-bungarotoxin (*Bungarus* spp.), taipoxin (*O. scutellatus*); *Other toxins*: botulinum toxin, tetanus neurotoxin. 2. **Voltage-gated calcium channel**: *Snake toxins* : calciseptine (*Dendroaspis* spp.), beta- bungaratoxin (*Bungarus* spp.); *Other toxins*: omega-conotoxin (marine snail, *Conus* spp.); *Disease states*: Lambert-Eaton myaesthenic syndrome. 3. **Pre-synaptic membrane**: *Snake toxins*: phospholipase A2 toxins. 4. **Pre-synaptic ACh receptor**: *Snake toxins*: candoxin (*Bungarus candidus*); *Other toxins*: curare; *Pharmacological substances* : non-depolarising blocking drugs (atracurium). 5. **Voltage-gated potassium channels**: *Snake toxins*: dendrotoxins (*Dendroaspis* spp.); *Disease states*: neuromyotonia, Isaacs' syndrome; *Pharmacological substances*: magnesium sulphate, aminoglycosides. 6. **Acetylcholine**: Lysis by exogenous acetylcholinesterase in *snake venom*: cobra venom (*Naja* spp.). 7. **Acetylcholinesterase**: Inhibitors of endogenous AChE in *snake venom*: fasiculins (*Dendroaspis* spp.). 8. **Post-synaptic ACh receptors**: *Snake toxins*: alpha-bungaratoxin (*Bungarus* spp.), candoxin (*B. candidus*), azemiopsin (*A. feae*), waglerin (*T. wagleri* ); *Other toxins*: alpha-conotoxin (marine snail, *Conus* spp.); *Disease states*: myasthenia gravis; *Pharmacological substances*: depolarising blocking agents (e.g., succinylcholine), non-depolarising blocking drugs (e.g., atracurium). 9. **Voltage-gated sodium channels**: *Snake toxins*: crotamine (*Crotalus* spp.); *Other toxins*: pompilidotoxin (wasps), delta-conotoxin (*Conus* spp.), tetradotoxin (pufferfish).

The peripheral neuromuscular weakness after snakebite results from defective neuromuscular junction (NMJ) transmission. It is pertinent to briefly review the current knowledge on NMJ transmission and neuromuscular block, to better understand the effects of snake venom at the NMJ [97]–[104] (Figure 1).

## Neuromuscular Transmission and Neuromuscular Block

At the pre-synaptic level, the motor nerve axon terminal is responsible for the synthesis, packaging, transport, and release of the neurotransmitter acetylcholine (ACh). Release of ACh in response to an incoming nerve action potential is triggered by the opening of voltage-gated calcium channels and the influx of calcium ions. Increased intracellular calcium concentration triggers a cascade of events that leads to the formation of a fusion complex made up of SNARE (Soluble N-ethylmaleimide-sensitive-factor Attachment REceptor) proteins, which enables fusion of ACh vesicles to the nerve terminal membrane and ACh release [98]–[102], [104]. Nicotinic acetylcholine receptors (nAChRs) at the nerve terminal (pre-synaptic neuronal autoreceptors -α3β2) facilitate release of increasing quantities of ACh, by mobilising ACh vesicles from a reserve pool to a releasable pool, in response to high frequency stimulation via positive feedback systems [98]–[100]. Interference with neuromuscular transmission at a pre-synaptic level can occur at voltage-gated calcium channels (e.g., Lambert Eaton myasthenic syndrome), SNARE proteins (e.g., botulism), potassium channels (e.g., neuromyotonia), or at the neuronal nAChRs.

ACh released from the nerve terminal diffuses rapidly across the synaptic cleft. Degradation of ACh at the synaptic cleft by acetyl cholinesterase (AChE) is necessary for the termination of its action.

At the post-synaptic level, ACh binds to muscle nAChRs (adult or mature type—α1β1εδ) on the post-synaptic membrane. nAChRs are ligand-gated ion channels, and their activation by ACh leads to an influx of sodium and calcium cations, accompanied by efflux of potassium ions through potassium channels, and produces an end-plate potential. If adequate ACh is released, this end-plate potential is propagated by the opening of sodium channels along the perijunctional zone and muscle membrane and initiates calcium release and muscle contraction [98]–[100], [102].

Neuromuscular block at the post-synaptic level is classified into non-depolarising and depolarising types. Depolarising neuromuscular blocking agents (NMBAs) (such as suxamethonium) bind irreversibly to the post-synaptic muscle nAChRs, and produce a non-competitive block, which is not reversed by acetyl cholinesterase inhibitor drugs (AChEIs). Depolarising NMBAs initially produce excessive depolarisation [97], which can be seen as muscle fasciculations [102]. This is followed by secondary changes responsible for muscle paralysis such as receptor desensitisation, inactivation of and blockage of voltage-gated sodium channels, and alterations in ion permeability of the membranes [97], [98]. Non-depolarising NMBAs (such as curare and its derivatives—d-tubocurarine, pancuronium, atracurium), in contrast, competitively inhibit ACh binding to the post-synaptic muscle nAChRs, and produce a competitive type of block. They repetitively associate with and dissociate from the ACh binding sites, rather than producing prolonged binding, and therefore can be displaced by ACh [97]. Blockade, therefore, can be reversed by AChEIs (such as edrophonium, neostigmine, and pyridostigmine) which act by increasing the available ACh at the synaptic cleft. The nAChR has two ligand binding sites, and both must be simultaneously occupied by ACh for the receptor to be active. The occupation of a single binding site by one molecule of a NMBA would therefore effectively “block” the receptor [98]–[100], [102]. Non-depolarising NMBAs, however, in addition have been shown to produce pre-synaptic effects by binding to the pre-synaptic, neuronal nAChRs (α3β2) [97]–[99], [105], [106], and this finding has challenged the traditional simplistic concept of pre-synaptic and post-synaptic block. Neurophysiologically, this dual effect is reflected by the combination of reduction in twitch amplitude (due to blockade of post-synaptic muscle nAChRs) and fade of the twitch height responses on repetitive (train-of-four or tetanic) stimulation (due to blockade of pre-synaptic neuronal nAChRs) [97], [98], [100]. Depolarising NMBAs, in contrast, only produce reduction in the twitch amplitude, but do not produce the tetanic or train-of-four (TOF) fade [98]–[100]. Non-depolarising block also produces a characteristic post-tetanic potentiation following high frequency (tetanic) stimulation [99], [100], [102], [107]. NMBAs are known to impair NMJ transmission by several additional effects on the nAChRs, without binding to the receptor binding sites. These include alteration of receptor dynamics, desensitization, and channel blockade [102].

## Snake Venom Toxins and Neuromuscular Block (Table 1, Figure 1)

**Table 1:** Summary of some key animal studies with individual snake neurotoxins.

Toxin	Authors; year; [reference]	Study description	Pathological change	Physiological/clinical effects
**Beta-bungarotoxin**	Dixon & Harris; 1999; [108]	In vitro–isolated nerve-muscle preparation (phrenic nerve-hemidiaphragm) in mice; In vivo nerve-muscle preparation in rats (sciatic nerve-soleus muscle; e/m: labeling of AChR, synapatophysin, or axonal neurofilament)	1) Depletion of synaptic vesicles (e/m: loss of synpatophysin immunoreactivity); 2) Destruction of motor nerve terminal (e/m: mitochondrial damage, Schwaan cell processes invading synaptic cleft); 3) Degeneration of axons (staining for anti-neurofilament antibodies): denervation starts at 3 h, 90% by 6 h, and complete by 24 h; 4) Reinnervation (by anti-synaptophysin labeling, labeling for axonal neurofilament): all NMJs reinnervated by 5 days, stable by 7 days, 90% by 14–21 days, reinnervation with multiple collateral innervation	Early onset paralysis—initial facilitation (maximal at 30 min), followed by irreversible failure of NMJ transmission (max. at 210 min)
**Beta-bungarotoxin**	Prasarnpun et al.; 2004; [117]	Rat phrenic nerve-hemidiaphragm	-	NMJ transmission failure—lag phase of 20–60 min, complete failure by 120–240 min
**Beta-bungarotoxin**	Prasarnpun et al.; 2005; [109]	Rat soleus muscle; e/m: NMJs and nAChRs identified; synaptic proteins (synaptophysin, SNAP-25, and syntaxin) labeled; sodium channels labeled; axon counts	1) 3–6 hours: depletion of synaptic vesicles, mitochondrial damage, transient upregulation of voltage-gated sodium channels, reduction in immunoreactivity of synaptic proteins; 2) Degeneration of terminal boutons, with isolation from post-synaptic membrane by Schwann cell processes, and withdrawal from synaptic clefts; denervation complete by 12 h; 3) Reinnervation starts at 3 days, and complete by 7 days. Progressive increase in the immunoreactivity of SNARE proteins: 75% by 7 days; 4) Persistent axonal loss at 6 months	Flaccid paralysis by 3 h; Return of function starting by 3 days, and complete by 7 days
**Alpha-bungarotoxin**	Lee et al.; 1977; [106]	In vivo cat sciatic nerve-tibialis anterior preparation	-	Gradual onset NMJ block—50% block in 30–60 min; No fade with tetanic or train-of-four stimulation; Post-tetanic facilitation
**Taipoxin, notexin**	Cull-Candy et al.; 1976; [111]	Isolated mouse phrenic nerve-hemidiaphragm preparation	Nerve terminal damage—depletion of synaptic vesicles, axoplasmic vacuoles, mitochondrial change, axolemmal indentations	NMJ block—Initial latency 40–60 min; maximal 110–120 min
**Notexin, taipoxin**	Harris et al.; 2000; [118]	In vivo rat soleus muscle; e/m: labeling of AChR and axonal neurofilament	1) Nerve terminal degeneration (depletion of synaptic vesicles, mitochondrial damage): start at 1 h, 70% by 24 h; 2) Axonal degeneration; 3) Reinnervation start at 2–3 days, 88% by 5 days, complete by 21–28 days; 4) Abnormal collateral innervation persistent at 9 months	-
**Crotoxin**	Hawgood et al.; 1977; [233]	Isolated mouse phrenic nerve-hemidiaphragm preparation	Inhibit quantal release of ACh at nerve terminal	-
**Candoxin**	Nirthanan et al.; 2002; [135]; 2003; [134]	Rat tibialis anterior muscle; mouse phrenic nerve-hemidiaphragm; binding to muscle nAChRs	-	Non-depolarising post-synaptic block; rapid onset; reversible with AChEIs; significant TOF fade

Traditionally it has been considered that snake venom toxins cause two types of neuromuscular blockade, pre-synaptic and post-synaptic; but this view may be oversimplistic and needs to be reviewed in view of the recent insights into neuromuscular transmission and descriptions of different patterns of neurotoxicity. Much of the current understanding of neurotoxicity has come from animal studies using purified individual toxins.

The pre-synaptically active neurotoxins (beta-neurotoxins—mostly neurotoxic phospholipase A_2_ toxins, PLA2s) bind to the motor nerve terminals, leading to depletion of synaptic ACh vesicles, impaired release of ACh, and later, degeneration of the motor nerve terminal [108]–[111]. They produce neuromuscular block that occurs in three phases: an immediate depression of ACh release, followed by a period of enhanced ACh release, and then complete inhibition of NMJ transmission [108], [112]–[116]. The effects on neuromuscular transmission develop following a latency period of 20–60 minutes [111], [114], [117]. The binding of pre-synaptic toxins to the nerve terminal is irreversible [109], [111]. Clinical recovery is slow as it is dependent on regeneration of the nerve terminal and formation of a new neuromuscular junction [109], [110]. Hence, patients with respiratory failure may need respiratory support for a longer period before spontaneous breathing can resume [108], [110], [113], [114], [118]. Treatment with antivenom or AChEIs is unlikely to be effective in pre-synaptic toxicity [108], [109], [114], [118], and incomplete recovery and delayed effects are more likely [108].

Pre-synaptic toxins are best illustrated by beta-bungarotoxin (b-BuTX) of kraits (*Bungarus* spp.) which predominantly has potent PLA2 enzymatic activity. Dixon and Harris (1999) first highlighted the significance of denervation in producing the treatment-resistant paralysis in krait bite [108]. They showed that beta-bungarotoxin produces pre-synaptic toxicity characterized by depletion of synaptic vesicles, destruction of motor nerve terminals, and axonal degeneration followed by reinnervation [108]. Prasarnpun et al. (2004, 2005) [109], [117] showed that beta-bungarotoxin produced calcium influx through voltage-gated calcium channels and increased release of ACh via SNARE-complex dependent mechanisms leading to depletion of synaptic vesicles. They were able to demonstrate the correlation between pathological changes and the neuromuscular transmission failure induced by beta-bungarotoxin [109]. Rat muscles inoculated with beta-bungarotoxin were paralysed within 3 hours. This was associated with loss of synaptic vesicles, mitochondrial damage, transient upregulation of voltage-gated sodium channels, and a reduction in immunoreactivity of SNARE proteins (synaptophysin, SNAP-25, and syntaxin). Between 3 and 6 hours after inoculation, nerve terminals showed evidence of degeneration. These included degeneration of terminal boutons, their isolation from the post-synaptic membrane by Schwann cell processes, and withdrawal from synaptic clefts. By 12 hours, all muscle fibres were denervated. Reinnervation began at 3 days with the appearance of regenerating nerve terminals, a return of neuromuscular function in some muscles, and a progressive increase in the immunoreactivity of SNARE proteins. Full recovery occurred at 7 days [109]. Harris et al. (2000) showed that taipoxin (from taipans, *Oxyuranus* spp.) and notexin (from the Australian tiger snake, *Notechis scutatus*) had effects similar to beta-bungarotoxin [118]. They suggested that all pre-synaptically active PLA2s produce similar effects [118].

Although the molecular basis of pre-synaptic toxicity induced by the PLA2s is still not completely understood [119]–[127], more recent studies have added significantly to our current knowledge [119]–[133]. They have shown that PLA2s from snake venom neurotoxins produce similar but complex effects on the pre-synaptic nerve terminal. These include entry into nerve terminals after binding to specific receptors on the pre-synaptic membrane, morphological changes such as nerve terminal bulging, changes in mitochondrial morphology and permeability, increase in cytosolic calcium levels, changes in expression and interactions of SNARE proteins, increased vesicle fusion and neurotransmitter release, and impaired vesicle recycling. Montecucco and colleagues have shown that the effects produced by four different snake venom PLA2s (beta-bungarotoxin, taipoxin, notexin, and textilotoxin) were similar, suggesting a similar mechanism of action for pre-synaptic neurotoxins. Hydrolysis of the phospholipids of the pre-synaptic membrane and membrane destabilization by the products of hydrolysis are likely to be key drivers in this process [122]–[124], [127], [131], [132].

The post-synaptically active neurotoxins (alpha-neurotoxins) bind to the post-synaptic muscle nAChRs. Alpha-neurotoxins belong to the group of “three-finger toxins” (3FTXs) characterized by a shared toxin structure resembling three outstretched fingers of a hand [134]–[137]. They are classified into three main groups—long-chain, short-chain, and non-conventional alpha-neurotoxins [134]–[137]. They resemble the action of d-tubocurarine (dTC), and are therefore called “curare-mimetic” neurotoxins. dTC classically produces a reversible, non-depolarising post-synaptic block by competitive inhibition of ACh binding to the muscle nAChR [97]. It also inhibits the pre-synaptic neuronal nAChRs, producing the characteristic TOF or tetanic fade. However, there can be significant variations in the effects of the so-called “curare-mimetic” neurotoxins on the post-synaptic nAChR. Some toxins (e.g., alpha-cobratoxin) have been shown to produce a competitive, non-depolarising type of post-synaptic blockade similar to dTC [138], [139]. In this type of toxicity, antivenom may facilitate dissociation of toxin from the ACh receptor and accelerate recovery [11], [110], and a clinical response to AChEIs, similar to myasthenia, is more likely [140]. Most of the alpha-neurotoxins, however, bind almost irreversibly to the post-synaptic nAChRs, even though they produce a non-depolarising type of block [106], [134], [136]. Their action, therefore, is not readily reversible by antivenom or AChEIs. These include most of the long-chain 3FTXs such as alpha-bungarotoxin.

Lee et al. (1977) showed that alpha-bungarotoxin (a-BuTX) produced a pure post-synaptic, non-depolarising, but almost irreversible neuromuscular blockade [106]. This was characterized by slow onset, persistent and dose-dependent progression, lack of recovery for a long period, and lack of sustained reversibility to AChEIs. Post-tetanic facilitation was prominent. Tetanic and TOF fade were not seen, and therefore this differed from the type of block seen with d-tubocurarine [106]. The lack of tetanic and TOF fade is attributed to the failure of alpha-bungarotoxin to block the pre-synaptic neuronal nAChRs [97]–[99].

The recent insights into NMJ transmission have enabled better and more comprehensive characterization of the more recently described toxins. Candoxin, a novel toxin isolated from the venom of the Malayan or blue krait (*Bungarus candidus*), is a non-conventional 3FTX with structural similarities to alpha-bungarotoxin [134]–[136]. However, in contrast to the nearly irreversible blockade produced by alpha-bungarotoxin, candoxin produces a readily reversible block of the post-synaptic nAChR. In addition, candoxin also inhibits the pre-synaptic, neuronal AChRs and produces tetanic and TOF fade on rapid repetitive stimulation [134], [136].

Although reversibility of blockade would be of crucial importance in the success of therapeutic interventions, what determines reversibility seems unclear. Low receptor binding affinity and a short polypeptide chain length of the toxin molecules have been postulated as likely reasons, but it is more likely that substitution of amino acid residues in regions that interact with the AChR may be responsible [134], [136].

Some snake venom toxins interfere with NMJ transmission through various other mechanisms. Some pre-synaptic toxins, such as the dendrotoxins from venoms of the Eastern green mamba (*Dendroaspsis angusticeps*) and the black mamba (*D. polylepis*), enhance ACh release from the nerve terminals by inhibiting potassium channels and produce a neuromuscular block similar to depolarising block [114], [141]. A different type of toxin from *D. angusticeps* acts as an AChE inhibitor, thus increasing the availability of ACh at the NMJ. They have been named fasciculins due to their effect of producing generalized, long-lasting fasciculations [136], [142]–[144].

## Snake Venom and Neuromuscular Block

Snake venoms do not contain a homogenous single toxin, but are complex cocktails of enzymes, polypeptides, non-enzymatic proteins, nucleotides, and other substances, many of which may have different neurotoxic properties [91], [112], [113], [116], [134], [145], [146] (Table 2). The studies of Chang and others of the Chinese (or formerly Formosan) cobra (*Naja atra*) venom (1966, 1972) [138], [139] highlighted the complexity of multiple actions of different neurotoxins in the same venom. They demonstrated that the main neuromuscular blocking effect was due to cobrotoxin, which produced a curare-like non-depolarising, competitive post-synaptic block, which was antagonised by neostigmine. It had no effect on nerve conduction. However, the venom also contained cardiotoxin, which interfered with axonal conduction and produced muscle depolarisation [138], [139].

**Table 2:** Some examples of toxin diversity in snake venom.

Snake type	Toxin	Species	Type of toxin	Neurotoxic effects	References
**Cobra (** ***Naja*** ** spp.)**	Alpha-cobratoxin	*N. kaouthia; N. siamensis*	Long-chain alpha-neurotoxin (3FTX)	1) Bind to post-synaptic muscle nAChRs—produce reversible, non-depolarising block; 2) Bind to neuronal α7 nAChRs	[136], [137], [234]
	Cobrotoxin	*N. atra*	Short-chain alpha-neurotoxin (3FTX)	Post-synaptic non-depolarising block	[138], [139]
	Cardiotoxin	*N. atra*	3FTX	Blocks axonal conduction, cytotoxicity	[138], [139]
	Toxin-alpha	*N. nigricollis*	Short-chain alpha-neurotoxin (3FTX)	Post-synaptic non-depolarising block	[136]
	“Weak toxin,” WTX	*N. kaouthia*	Non-conventional alpha-neurotoxin (3FTX)	1) Bind to post-synaptic muscle nAChRs—produce irreversible, non-depolarising block; 2) Bind to neuronal α7 nAChRs	[136], [235]
**Krait (** ***Bungarus*** ** spp.)**	Alpha-bungarotoxin	*B. multicinctus*	Long-chain alpha-neurotoxin (3FTX)	Bind to post-synaptic muscle nAChRs—produce irreversible, non-depolarising block	[106], [136]
	Beta- bungarotoxin	*Bungarus* spp.	Phospholipase A2	Pre-synaptic block	[108], [109], [117]
	Kappa-bungarotoxin	*B. multicinctus*	Kappa-neurotoxin (3FTx)	Block neuronal nAChRs in autonomic ganglia	[137], [149], [150], [236], [237]
	Candoxin	*B. candidus*	Non-conventional alpha-neurotoxin (3FTX)	1) Bind to post-synaptic muscle nAChRs—produce reversible, non-depolarising block; 2) Bind to neuronal α7 nAChRs	[134]–[136]
**Russell's viper (** ***Daboia*** ** spp.)**	Phospholipase A2 activity	*D. russelii*	Phospholipase A2	Pre-synaptic block	[147], [148]
	Daboia Neurotoxin-1 (DNX-1)	*D. russelii*	Short-chain neurotoxin	Post-synaptic block	[146]
	Viperotoxin-F	*D. russelii*	Phospholipase A2	Pre-synaptic block	[113], [238]
**Mamba (** ***Dendroaspis*** ** spp.)**	Dendrotoxins—alpha, delta, I, K	*D. angusticeps, D. polylepis*	3FTX	Block neuronal voltage-gated potassium channels—pre-synaptic +/−post-synaptic effects	[141], [200]
	Fasciculins	*D. angusticeps, D. polylepis*	3FTX	Inhibit AChE	[143], [144]
	Muscarinic toxins	*D. angusticeps*	3FTX	Muscarinic effects by binding to muscarinic AChRs	[136], [142], [204]
	Calciseptine	*D. polylepis*		Inhibit voltage-gated calcium channels	[239]
**Rattlesnake (** ***Crotalus*** ** spp.)**	Crotoxin	*C. durissus*	Phospholipase A2	1) Pre-synaptic block; 2) Post-synaptic effect by desensitization of nAChR	[116], [228], [233], [240], [241]
	Mojave toxin	*C. scutulatus*	Phospholipase A2	Pre-synaptic ion channel blocker	[116], [170], [242]

Characterization of new toxins continues to add to the rich diversity of snake venom, and many types of venom are now known to contain both pre- and post-synaptically active toxins. For example, a post-synaptic toxin (DNTx-I—Daboia Neurotoxin 1) has been isolated from the venom of Russell's viper (*Daboia russelii*) [146], in addition to the well-known pre-synaptic PLA2 toxin [147], [148]. Venom of kraits (*Bungarus* spp.) consists of several different types of neurotoxins. In addition to the alpha-bungarotoxin (post-synaptic block) and beta-bungarotoxin (pre-synaptic block) already described, it also contains kappa-bungarotoxin which binds to the neuronal nAChR at the post-synaptic level in central cholinergic synapses in autonomic ganglia [109], [149], [150].

Experimental data on physiological, pathological, and ultrastructural changes due to snake neurotoxins are derived from studies in animal models, in vitro nerve-muscle preparations, or preparations of nAChRs. However, such laboratory data may not accurately reflect the effects of snake venom in humans. It is known that the effects of envenoming can vary depending on the bitten species, and this may be due to the snake's prey preferences.

For example, candoxin from the Malayan or blue krait (*B. candidus*), which feeds mainly on rodents and reptiles, preferentially binds to murine nAChRs rather than to those of chick origin [135]. Irditoxin from the venom of the brown tree snake (*Boiga irregularis*) shows taxa-specific lethal toxicity to birds and lizards, but not toward mice. In vitro studies showed that it produced potent post-synaptic toxicity similar to alpha-bungarotoxin at avian NMJs, but not in mammalian NMJs [151]. Waglerin from Wagler's pit viper (*Tropidolaemus wagleri*) binds more tightly to mouse nAChRs than to those from rats or humans [152]. Similarly, interspecies differences in sensitivity of nerve-muscle preparations to pre-synaptic snake toxins have been well documented [113].

Furthermore, some in vitro studies of toxins have been done on nAChRs of αβγδ type, which is the foetal type of nAChR, in contrast to the adult (or mature) type (αβεδ) of nAChR normally expressed in the NMJ [135]. It is known that the two different types of receptors have different opening times and speeds of ion conductance [97], [98]. It is likely that the effects of toxins on the two different types of receptor, and therefore the in vivo effects on humans, may be different to what may be observed in the laboratory. In this context, it is interesting to note that waglerins from Wagler's pit viper (*Tropidolaemus wagleri*) and azemiopsin from Fea's viper (*Azemiops feae*) have shown specificity toward the ontogenetic state of the nAChR, with higher binding affinity to the adult (or mature) form than the foetal form [152]–[155].

## Clinical Manifestations

### Acute Neuromuscular Paralysis (Figures 2, 3, 4)

**Figure 2:**
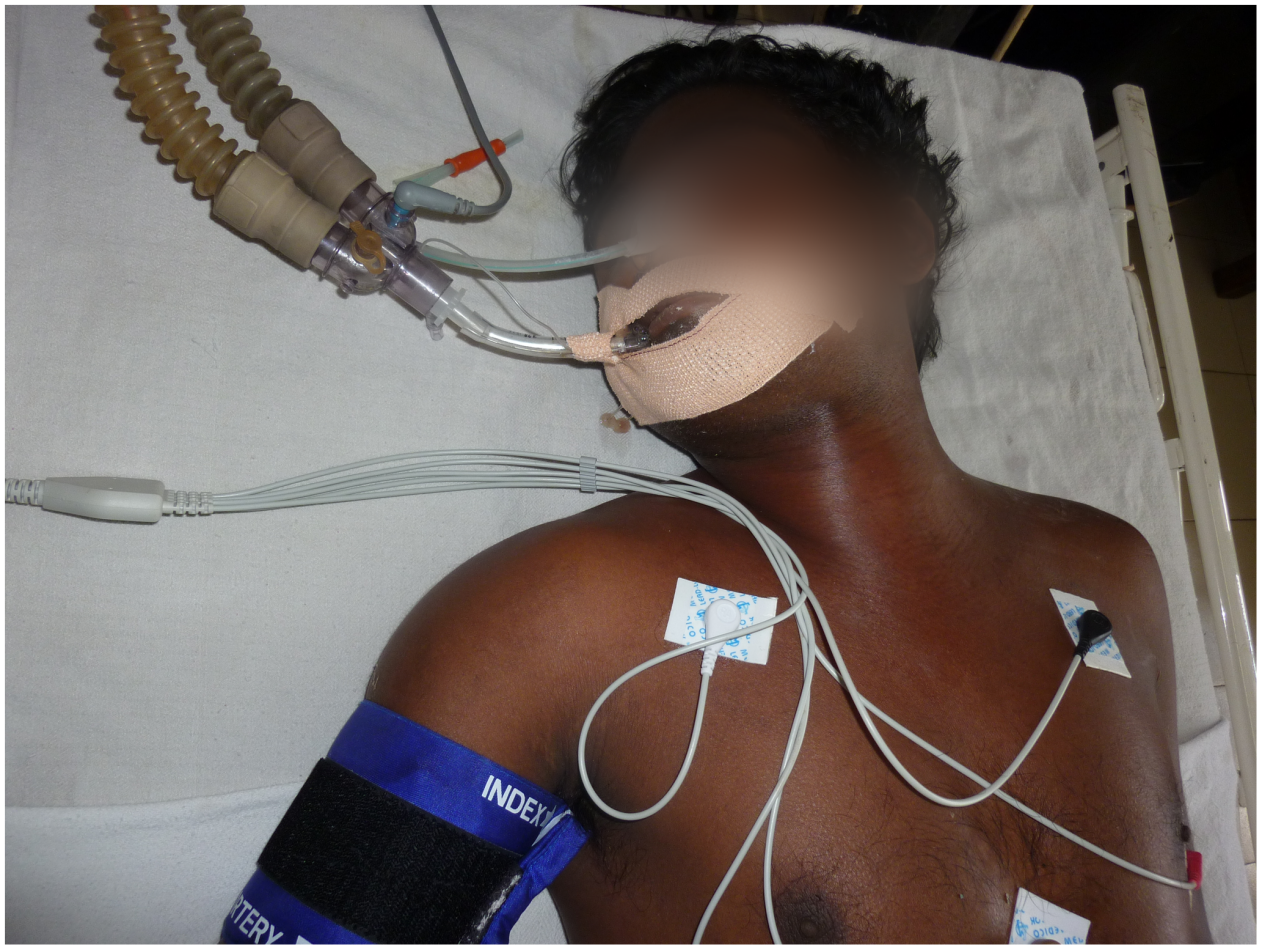
Respiratory paralysis in neurotoxic envenoming. Sri Lankan patient with severe neurotoxicity and respiratory paralysis being ventilated following a cobra (*Naja naja*) bite. (Photograph courtesy of Prof. S. A. M. Kularatne, University of Peradeniya, Sri Lanka. The purpose of the photograph has been explained to the patient, and consent obtained for potential publication.)

**Figure 3:**
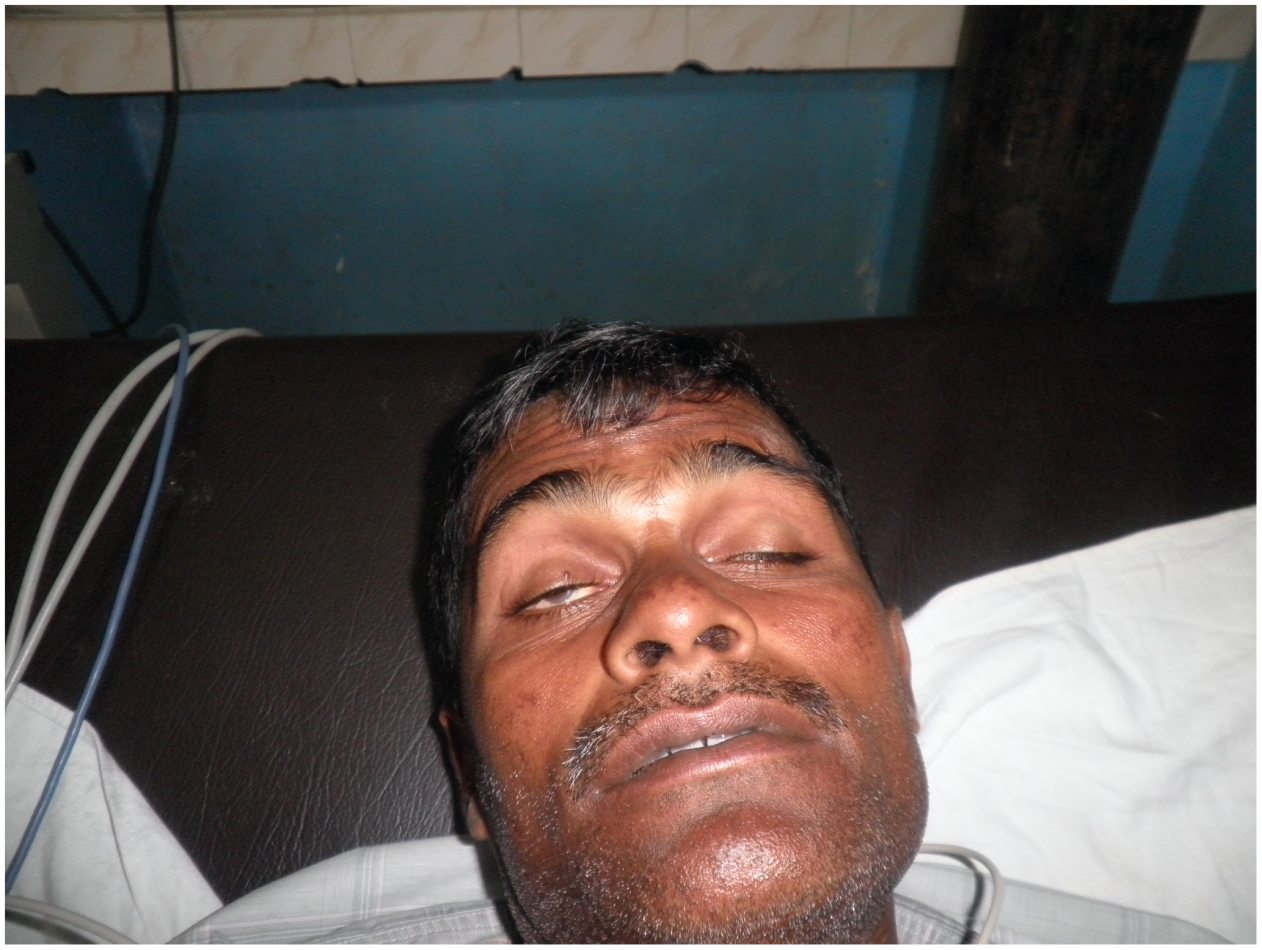
Bilateral ptosis and facial weakness in neurotoxic envenoming. Sri Lankan patient with bilateral ptosis and facial weakness following a Krait (*Bunagrus caeruleus*) bite. (Photograph courtesy of Prof. S. A. M. Kularatne, University of Peradeniya, Sri Lanka. The purpose of the photograph has been explained to the patient, and consent obtained for potential publication.)

**Figure 4:**
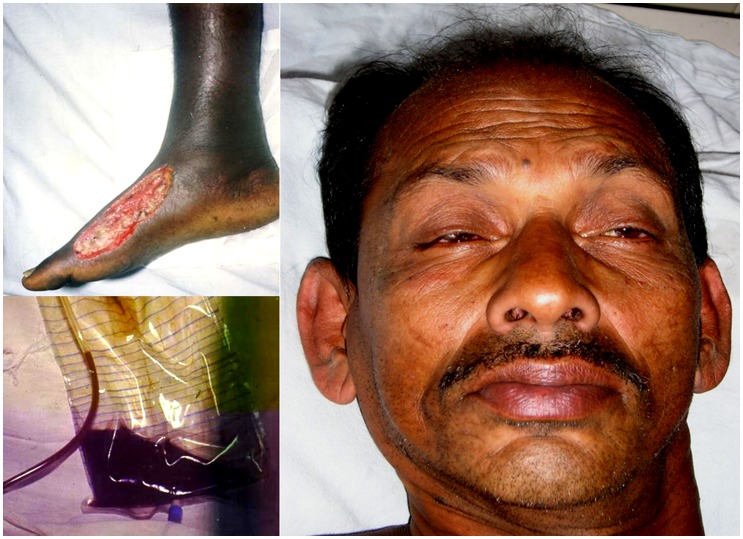
Neurotoxicity in Russell's viper envenoming. Sri Lankan farmer with Russell's viper (*Daboia russelii*) envenoming: tissue necrosis at bite site, haematuria, and bilateral ptosis. (Photographs courtesy of Prof. S. A. M. Kularatne, University of Peradeniya, Sri Lanka. The purpose of the photographs has been explained to the patient, and consent obtained for potential publication.)

Reported prevalence rates of neuromuscular weakness vary between series, and with different snake species and different study settings. As seen from above, each toxin can have a multitude of effects, venom of one snake species is a mix of multiple toxins, and venom composition varies between species of same genus. In addition, intraspecies variations in venom compositions are well known. It is therefore not surprising that wide variation is seen in the neurotoxic effects.

Possible methodological differences and lack of uniformity in description contribute to the large variation between studies, and make interpretation of data from different studies difficult. Ptosis is reported in between 70–93% of patients in most series, and extraocular muscle weakness in 68–82% [9]–[11], [42], [73], [75], [156]. Respiratory muscle weakness is reported in 27–87% [9]–[11], [25], [42], [45], [156]. Case fatality rates with neurotoxic envenoming again show wide variation, usually ranging between 4–11% [10], [11], [14], [20], [25], [26], [31], [42], [94], [156], but rates as high as 37% have been reported [20]. Variations in case fatality are likely to be influenced by many factors including intensity of staff observation, development of respiratory failure, and availability of ventilation. Intensive care units are more likely to admit more severe patients with respiratory involvement [11], [26], [94], [157], and therefore may have higher mortality rates than less-biased samples. Delays in accessing ICU care, and lack of adequate facilities for optimal care in resource-limited areas where snakebite is common, also would contribute to higher mortality [20].

The envenoming snake species is highly likely to influence the clinical presentation and outcome, but many studies have considered together bites from different snake species [9], [14], [26], [37], [94], [156]–[158]. Such differences are perhaps unavoidable as confirming the identity of the envenoming snake is often difficult. Only a few studies have reported snake identification by detection of venom antigens [21], [30], [31], [42], [45], [69], [159]. A reasonably representative picture of neurotoxicity with different snakes can only be obtained from studies with larger numbers of unselected patients admitted to general care units, accurate species identification, and a focus on bites by a single type of snake (see Table 3).

**Table 3:** Summary table of some key studies with descriptions of neurotoxicity.

Author; year; type of snake; [reference]	No. of patients	Ptosis frequency %	Extraouclar weakness frequency %	Limb weakness frequency %	Neck muscle weakness frequency %	Weakness, onset (median/mode; range)	Weakness, max. (median/mode; range)	Weakness, start recovery (median/mode; range)	Weakness, complete recovery (median/mode; range)	Resp. paralysis/ventilation frequency %	Ventilation duration (median/mode; range)	Duration of hospital stay (median/mode; range)	Case fatality %
Watt et al.; 1988; *N. philippinensis*; [31]	39	87	87	97.4	N/A	Median: 60 min (3 min–24 h)	N/A	N/A	N/A	45	N/A	N/A	5.1
Kularatne; 2002; *B. caeruleus*; [10]	210	70	N/A	64	60	N/A	N/A	N/A	8–9 d	48	Mode: 2 d (12 h–29 d)	N/A	7.6
Ariaratnam et al.; 2008; *B. caeruleus*; [25]	88	N/A	N/A	N/A	N/A	½ h–4 h	N/A	N/A	N/A	64	Mean: 5 d (18 h–16 d)	N/A	6
Lalloo et al.; 1995; *O. scutellatus*; [42]	166	85.4	76.6	57.9	N/A	Median: 6 h	N/A	Median: 48 h	N/A	36.7	88 h (6–500 h)	N/A	4.3
Phillips et al.; 1988; *D. russelii*; [69]	23	77	82	0	0	Mean: 2.3 h (1/2 h–7 h)	N/A	Mean: 2.7 d (1–4 d)	1–8 d	0	0	N/A	N/A
Kularatne; 2003; *D. russelii*; [75]	336	78	64	N/A	22	N/A	N/A	N/A	Mean: 3 d (1–5 d)	2.4	N/A	Mode: 4 d	2.6

(N/A – not available).

### Respiratory Muscle Weakness

Many patients with neurotoxicity develop ptosis and extraocular muscle weakness, but only a few will develop respiratory muscle weakness. Factors that determine development of respiratory muscle weakness in some patients are not clear. The traditionally held view that it is related to the dose of venom and the severity of envenoming, perhaps modified by antivenom therapy, has not been adequately addressed. The possibility that distinct patterns of neuromuscular weakness exist in snake envenomation has not been studied. A parallel may be drawn with myasthenia gravis where two forms of weakness, ocular and generalized, are well known. Extraocular muscles are developmentally, histologically, ultrastructurally, immunologically, metabolically, and functionally different to other skeletal muscle groups [160]–[166]. They have a mixture of several different fibre types, including singly innervated fast-twitch fibres and multiply innervated slow-twitch fibres [160], [161], [165]. Their NMJs are different, with lower AChR densities and lower quantal ACh contents [165]. Both adult (αβεδ-) and foetal (δβγδ-) isoforms of nAChR are expressed in adult extraocular muscles, unlike in other skeletal muscles [163], [165], [166]. It is not surprising that they are involved differently in various pathological processes. They are preferentially affected in some diseases such as myasthenia and chronic progressive ophthalmoplegia, and selectively spared in Duchenne muscular dystrophy and amyotrophic lateral sclerosis [161], [163], [164]. There needs to be further study at the molecular level of the effect of different snake venom neurotoxins on development of respiratory muscle weakness.

Ptosis and extraocular weakness are commonly reported in Sri Lankan Russell's viper envenoming [9], [69], [72], [73], [75], but reports of respiratory involvement are sketchy [9], [75].

It is known that different toxins have different affinities to the two isoforms of muscle nAChRs [152]–[155] (as described earlier), but whether this can explain the different patterns of selective muscle group involvement needs further study.

The natural history of neurotoxic envenomation is likely to vary with the degree of envenoming and snake species, and between patients. There are little data on the natural course, as it can be affected by treatment. In a rare case series of 60 patients with envenoming by the many-banded krait (*Bungarus multicinctus*) in Vietnam for whom antivenom was not available, 87% needed mechanical ventilation for a mean of 8 days, the mean duration of the ICU stay was 12 days, and hospital mortality was 7% [11].

## Neurotoxicity, Type of Snake, and Possible Geographical Variation

There is a clear variation in the propensity of similar species of snakes to produce different patterns of neuromuscular weakness in different geographical locations. For example, the Philippine cobra (*Naja philippinensis*) produces more neurotoxicity and less local swelling [31] compared to other Asian cobras [21], [32], [38]. There are several reports of neurotoxicity due to envenoming by Russell's viper (*Daboia russelii*) in Sri Lanka and South India, in contrast to reports of bites by Russell's viper from other countries [7], [9], [68], [69], [72]–[75].

These geographical differences may be due, at least in part, to interspecies and intraspecies differences in venom compositions. The venom composition in Russell's viper in Sri Lanka (*Daboia russelii*) and South India (*D. russelii*) was found to be different from that found in Pakistan (*D. russelii*), Thailand (*D. siamensis*), and Taiwan (*D. siamensis*) [167]. However, even with similar venom compositions, the difference between clinical reports from Sri Lanka and India are striking. While neurotoxicity has been reported in a majority (∼80%) of Sri Lankan patients with Russell's viper envenoming [69], [73], [75], there are only isolated case reports from India [7], [74]. Although these differences may be attributed to poor reporting, a prospective case series of viper bites from India did not report any neurotoxicity [168]. In addition, there are several reports of fascinating regional variations in venom composition and potency from the same species (intraspecies variation) within the same country, e.g., Russell's viper (*Daboia russelii*) in India [147], [169], the Mojave rattlesnake (*Crotalus scutulatus*) in the United States [170], the asp viper (*Vipera aspis*) in France [76], [81], and tiger snakes (*Notechis scutatus*) in Australia [171].

## Neurophysiological Changes in Neuromuscular Paralysis

Surprisingly few human data are available on the acute neurophysiological changes after snakebite. The available data mainly examine the defective transmission at the neuromuscular junction, with evidence for both pre-synaptic and post-synaptic defects (see Table 4). Interpretation of the findings from these studies is difficult, as different methodologies have been used (e.g., different rates of repetitive stimulation). Several articles describe neurophysiological changes, but carry insufficient details of the neurophysiological assessments [172]. There are very little data on single-fibre EMG findings, which would best document defective NMJ transmission.

**Table 4:** Some human studies with neurophysiological findings in snake neurotoxicity.

Author; year; no. of patients; [reference]	Snake spp.	Neurophysiological findings	Interpretation
Watt et al.; 1986; n = 2 (out of 10); [35]	*N. philippensis*	Decremental response with 5 Hz RNS	Non-depolarising, competitive post-synaptic block
Singh et al.; 1999; n = 12; [23]	*B. caeruleus*	Reduction in CMAP amplitudes on motor nerve stimulation; decremental response to 3 Hz RNS	Both pre-synaptic and post-synaptic effects
Connolly et al.; 1995; n = 3; [44]	*O. scutellatus*	Reduction in CMAP amplitudes on motor nerve stimulation; decremental response to 5 Hz RNS with post-activation potentiation followed by exhaustion; blocking and increased jitter with single-fibre EMG	Pre-synaptic defect
Trevett et al.; 1995; n = 24; [45]	*O. scutellatus*	Reduction in CMAP amplitudes on motor nerve stimulation; reduction in SNAP amplitudes on sensory nerve stimulation; decremental response to 3 Hz RNS; post-tetanic potentiation followed by exhaustion	Pre-synaptic defect

(CMAP, compound muscle action potential; RNS, repetitive nerve stimulation; EMG, electromyography; SNAP, sensory nerve action potential).

## Treatment of Neuromuscular Paralysis in Snake Envenoming (Table 5)

### Antivenom in Neurotoxicity

**Table 5:** Summary of studies on interventions in neurotoxic envenoming.

Intervention	Author; year; [reference]	Snake spp.	No. of pts	Method	Outcome
**Antivenom**	Agarwal et al.; 2005; [94]	mixed	55—needing ventilation	Low-dose vs. high-dose antivenom	No difference between high and low doses
**Antivenom**	Ha et al.; 2010; [175]	*B. multicinctus*	81	Non-randomized, controlled trial (historical control)	Antivenom effective—reduces duration of weakness, ventilation, and ICU stay
**Antivenom vs. edrophonium**	Watt et al.; 1989; [30]	*N. philippensis*	8	Randomized, double-blind trial	Antivenom not effective; Edrophonium effective
**Antivenom; edrophonium**	Phillips et al.; 1988; [69]	*D. russelii*	23	Descriptive case series	Antivenom not effective; Edrophonium not effective
**Antivenom and neostigmine**	Anil et al.; 2010; [27]	*B. caeruleus*	54	Descriptive case series	Antivenom not effective; Neostigmine not effective
**Edrophonium**	Watt et al.; 1986; [35]	*N. philippensis*	10	Randomized, placebo-controlled, double-blind, cross-over trial	Edrophonium effective—with improvement in clinical and neurophysiological parameters
**Edrophonium and 3,4-DAP**	Trevett et al.; 1995; [46]	*O. scutellatus*	50	Placebo-controlled trial	No significant improvement with edrophonium or 3,4-DAP

There are many unresolved questions regarding the use of antivenom in snake envenomation–related neurotoxicity, and available reports show conflicting results. Differences in study methodology (species of snake, single snake type or “mixed bag,” presence of respiratory paralysis, severity of envenoming, delays to treatment) are likely to contribute significantly to the reported variations in response to treatment. In addition, such variations may well be related to the differences between pre-synaptic and post-synaptic types of toxin in snake venom, and also to the specificity of antivenom to the envenoming snake species. How much of the reported recovery may be attributable to spontaneous recovery is not clear, and there are a number of reports of recovery from neuromuscular paralysis without antivenom [11], [173], [174]. There are few well-documented reports of benefit with antivenom [18], [48]. Even in such reports, benefits have not been consistent and were seen only in some patients. In contrast, many of the well-documented case series report no benefit with antivenom in neuromuscular failure [10], [21], [27], [30], [42], [61]. However, several studies have observed improvement in neurotoxicity when antivenom had been administered very early [40], [42], [54], [69]. Antivenom cannot neutralise bound venom, and can be effective only if given early enough to neutralise circulating venom before it binds to target sites [42], [94]. It is likely that early administration is critical for success. Placebo-controlled randomized clinical trial data of antivenom in neurotoxicity are lacking. In a randomized double-blind trial in Philippine cobra (*Naja philippinensis*) envenoming, antivenom was not found to be effective [30]. However, in a recent non-randomized trial from Vietnam, antivenom was reported to be beneficial in patients with envenoming by the many-banded krait (*B. multicinctus*) [175]. A key limitation in this study was that patients given antivenom were compared with a group of patients treated during an earlier period for whom antivenom was not available (historical control). In experimental conditions, toxin-specific antibodies have been shown to reverse neurotoxic effects. Gatineau et al. (1988) reported that specific antibodies against *Naja nigricollis* toxin-alpha were able to reverse toxin binding to the AChRs and the resultant neuromuscular paralysis [176].

### Acetylcholinesterase Inhibitors (AChEIs, Anticholinesterases) in Neurotoxicity

Neuromuscular weakness, especially due to non-depolarising post-synaptic blockade, has similarities to myasthenia in pathophysiology, and it is theoretically plausible that AChEIs are effective in this type of neurotoxic envenoming. However, the evidence for benefit of AChEI is conflicting. This may well be due to the confounding effects of any natural recovery, co-administered antivenom, and different types of envenomation by different species.

There are several reports of benefit from AChEIs [18], [20], [30], [35], [51], [54], [177]–[181]. Some reports suggest that an edrophonium test (Tensilon test) can be used to predict the response to treatment with the longer-acting neostigmine [35]. Two small clinical trials have shown benefit with edrophonium, and both were in Philippine cobra (*Naja philippinensis*) envenoming [30], [35]. There are several reports of lack of benefit with AChEIs in envenoming by kraits [15], [21], [24], [27], taipans [46], and coral snakes [47].

It is likely that a good response to AChEIs is seen only in the competitive, reversible type of post-synaptic toxicity [51]. Reports of benefit with AChEIs in envenoming by snakes producing predominant pre-synaptic toxicity are unusual [18]. Similarly, antivenom is likely to be effective only in the competitive, reversible type of post-synaptic toxicity. Well-controlled clinical trials with proper snake identification are urgently needed to identify which patient groups will benefit from these potentially beneficial treatments, and equally importantly, to identify patients for whom they should not be given. Currently, it is routine practice to administer antivenom to all patients with neurotoxic envenoming, with little evidence of benefit, perhaps based on anecdotal reports of persistent neuromuscular problems in those not receiving antivenom [42]. Antivenoms used in developing countries are known to produce adverse reactions in 30–80% of patients [159], [182], and reactions can be seen in up to 25% even in developed countries [182]. Although symptoms are mild in most cases, severe systemic anaphylaxis may develop, and further understanding of their harm-benefit balance is important.

## Acute Neurotoxicity—Other Neurological Manifestations

Several other interesting acute neurological features have been reported after snake envenomation, which are likely to be direct neurotoxic effects. The mechanisms of many of these acute manifestations are not clear, and there has been no systematic study of these in a large series.

Myokymia has been reported mainly from the United States following rattlesnake (*Crotalus* spp.) envenoming, providing further evidence of variation in neurotoxicity with species and geographical differences [60]–[64], [66]. Respiratory failure developed in some patients who had myokymia involving the shoulders or chest, perhaps due to underlying diaphragmatic involvement [60]. Myokymia is believed to be due to a biochemical effect on axonal ion channels leading to increased peripheral nerve excitability [60], [64]. Crotamine in South American rattlesnake (*Crotalus* spp.) venom has been shown to act on voltage-gated sodium and potassium channels [183]–[189], and similar molecules may be responsible for the myokymia in envenoming by North American rattlesnakes (*Crotalus* spp.). Inhibition of pre-synaptic voltage-gated potassium channels is seen in neuromyotonia, which is an autoimmune disorder presenting with continuous fasciculations. It would be interesting to see whether a similar mechanism exists in myokymia due to rattlesnake envenoming [114].

There are several reports of central effects such as drowsiness, coma, and loss of brainstem reflexes following snakebite. Many of them are isolated case reports with poor snake identification [190]–[192]. Assessment of central effects due to direct neurotoxicity can be difficult, as similar effects can be produced by cerebral haemorrhage and ischaemia in snake envenoming, seen especially with viperid bites. Appropriate neuroimaging would be important to exclude these effects.

A large series of common krait envenoming has reported altered consciousness in 64% of patients, and deep coma in 17% [10]. Drowsiness was common among children with cobra bites [37]. Seizures have been noted in several reports [9], [73], [94]. Alterations in smell and taste have been reported in envenoming by several snake species [67], [193], [194], and whether these are central effects or due to peripheral cranial nerve involvement is not clear.

New studies shed light on the possible diverse effects of snake neurotoxins beyond the neuromuscular junction, and there are several reports of their actions on central nervous system neurones in animal studies; e.g., kappa-bungarotoxin is known to block central post-synaptic nAChRs [149], alpha-cobratoxin can produce central pain-relieving actions, probably via cholinergic pathways [195], beta-bungarotoxin affects neurotransmitter storage and release in central synaptosomes [196], [197], waglerin-1 inhibits GABAergic transmission [198], [199], and dendrotoxins have been shown to produce electrocortical convulsions, EEG discharges, and neuronal damage [200]–[203]. In addition, several muscarinic toxins have been identified from *Dendroaspis* spp. [142], [204]. While the pathological significance of these toxic effects in humans is not clear, these findings clearly demonstrate the possibility of neurotoxins affecting the central nervous system. There are several reports of snake neurotoxins interacting with the blood-brain barrier, which increase the likelihood of in vivo direct central neurotoxic effects [205]–[210].

Autonomic involvement, especially parasympathetic denervation effects, are reported in several case series and case reports, and almost all these reports are following krait bites [10], [11], [15], [18]. This is likely to be related to defective ACh transmission at parasympathetic nerve terminals, but the exact mechanisms have not been identified. Neurotoxins have been shown to bind to nAChRs in autonomic ganglia but the significance of this in humans is not clear [149], [150]. In addition, a few cases of acute neuropathy have been reported following envenoming by Russell's viper (*Daboia russelii*) [72], [74] and Eastern coral snake (*Micrurus fulvius*) [48].

## Delayed Neurological Manifestations

There are several reports of delayed neurological manifestations after snake envenomation. Some are reports of persistence of neurological deficits which first developed during the acute stage. Distinction from critical illness neuropathy and myopathy may be difficult when symptoms are first noticed soon after recovery from the acute phase, especially with a background of ventilation, ICU care, or sepsis [28], [211]. There are several other reports of neurological deficits developing at variable time points after recovery from the acute phase of envenoming. Some of the reports are confined to reporting of prolonged symptoms [11], and objective documentations with neurophysiological assessments are rare. In a series of 210 patients bitten by the common krait (*Bungarus caeruleus*), 38 patients had delayed neurological deficits. Fourteen of them had nerve conduction defects that lasted for 2 weeks to 6 months before complete recovery [10]. There are several reports suggestive of polyneuropathy after the acute phase of envenoming, with persistence of symptoms for several months [9], [65]. Several cases of possible Guillain-Barré syndrome (GBS) have been reported. One patient developed motor and sensory neuropathy 2 weeks after an unidentified snakebite and treatment with antivenom and tetanus toxoid. His clinical, biochemical, and electrophysiological features were suggestive of GBS [212]. Another report is of a patient who had acute neurotoxicity and respiratory arrest after a krait bite and developed quadriparesis 3 weeks later with elevated CSF protein and evidence of a sensorimotor axonal-type polyneuropathy [22]. However, GBS seems unlikely here as he had a coma with dilated pupils. Perhaps the most interesting report is by Neil et al. (2012) who describe a case of GBS after a bite by *Vipera aspis*. They have demonstrated a potential immunological basis for the syndrome, with cross-reactivity shown between glycosidic epitopes of venom proteins and neuronal GM2 ganglioside, without evidence of direct neurotoxicity of the venom [80].

There are few robust studies of long-term neurological effects. In the first detailed clinical and neurophysiological study of long-term neurological deficits, Bell et al. studied 26 asymptomatic survivors who had evidence of neurotoxicity during acute envenomation one year earlier [213]. Significant differences were noted in some neurophysiological parameters compared with controls. These included prolongation of sensory, motor, and F-wave latencies, and reduction of conduction velocities. The changes were more marked in the upper limbs than the lower limbs, suggesting a systemic effect related to envenoming rather than local neurological damage, as all cases in the study were bitten on the lower limb. No abnormalities were seen on repetitive nerve stimulation, indicating lack of residual deficits in neuromuscular junction transmission. Taken together, the results were suggestive of a non-length-dependent demyelinating-type polyneuropathy. The neurophysiological abnormalities were not typical of a toxin-mediated neuropathy, which usually would be associated with axonal damage. Interestingly, abnormalities in nerve conduction were only seen in those with presumed elapid bites [213].

The factors responsible for the causation of long-term neurological effects need further study. Persistent axonal damage due to neurotoxins, and delayed immune-mediated reactions to toxins or antivenom are possible explanations. There is also some experimental evidence for delayed neuropathic effects. In their report of beta-bungarotoxin–induced toxicity in rats, Prasarnpun et al. observed loss of myelinated axons at 6 months after inoculation [109].

## Discussion

Although the clinical manifestations of acute neuromuscular weakness with respiratory involvement are well recognised, it is surprising how many questions remain unanswered regarding neurotoxicity. This lack of clarity may at least partly be explained by the emerging evidence that has led to an increased understanding of neuromuscular transmission. This suggests that previously held traditional models of two different types of neurotoxicity (pre-synaptic or post-synaptic) are inadequate to explain all of the differences seen in symptom evolution and recovery, patterns of weakness, respiratory involvement, and responses to antivenom or AChEI therapy. For example, it is becoming clear that many of the post-synaptic toxins produce nearly irreversible binding, and long-lasting effects. The importance of the reversibility of post-synaptic toxicity, and the potential for blockage of pre-synaptic nAChRs by “post-synaptic” toxins after envenoming have not been addressed in adequate detail. This variability in toxicity may partly explain the differences in the pattern of envenomation by different species in different geographical regions, and it is highly likely that the presence of a number of different toxins in one venom also contributes. Detailed analysis of venoms from different snake species from different regions may help further elucidate these.

In addition to neuromuscular failure, several other interesting acute and delayed neurological manifestations have been described after snake envenomation, and there is very little understanding of their pathophysiological basis. These are further pointers to the diversity of the types of neurotoxicity produced by different snake species. There is no agreed time cut-off for classifying neurological manifestations into “acute” and “delayed/late.” There is a clear need for a uniform classification of delayed neurological manifestations. We propose that changes be classified as acute (onset within the first 2 weeks after snakebite, which may persist until late stages), delayed (onset within 2–8 weeks), and late (onset after 8 weeks of envenoming).

Improved case definitions are the key to a better understanding of neurotoxicity from different snakes. This can only be achieved by either the identification of dead snakes or the use of laboratory or near-patient detection of venom antigen. Further development of such techniques for developing countries where snakebites are common is vital to allow accurate and meaningful clinical descriptions of neurotoxicity.

Given the high morbidity and mortality, better treatment options are clearly needed in neurotoxic envenoming. There are several exciting reports of the use of plant extracts in the treatment of neurotoxicity [214]–[222]. Although promising, much more research is needed before these may become therapeutic options. Until such innovative treatments are available, much can be achieved by public health measures such as better education with emphasis on early hospitalization, improved availability of antivenom and intensive care facilities in areas where snakebite is common, and international collaborative efforts to develop such strategies in these resource-limited settings. Development of more effective and safer antivenoms including monospecific antivenoms and Fab fragments, and a better understanding of the cross-neutralisations possible with available antivenom, may help to optimize the use of antivenom in neurotoxicity [182], [223]–[226].

Given the lack of clarity over mechanisms of neurotoxicity, the lack of consensus on the value of antivenom or AChEI therapy in snake envenoming is not surprising. Conflicting reports of their efficacy are likely to reflect different mechanisms of neurotoxicity produced by different snake species, and potentially, variations in antivenom efficacy and time of administration. Models to predict type of toxicity, and a better understanding of the type of toxicity produced by different species, would perhaps enable better use of these treatment strategies. More data are needed on their efficacy, and may be obtained only from clinical trials in envenomation by different snake species. Electrophysiological studies may also be valuable in helping us to understand the complex processes in human neurological envenoming.

Snake neurotoxins have contributed significantly to our understanding of neuromuscular transmission and receptor function, and recent studies have highlighted many of their other properties, e.g., the ability to enter actively proliferating cells, nuclear localization, preferential binding in specific cell division phases, inhibition of apoptosis, anti-inflammatory and analgesic actions, and antimicrobial effects [183], [195], [227]–[232]. More research into these fascinating molecules and their diverse actions would not only help us improve management of neurotoxic envenoming, but may also enable their use as potential treatments for infections, cancer, and various neurological disorders.

**Key Learning Points**

1. Snake venoms are complex mixtures of different toxins, and each neurotoxin has diverse neurotoxic effects.
2. There is considerable geographical, interspecies, intraspecies, as well as possibly ontogenetic variation in neurotoxicity with snake envenoming.
3. Accurate identification of envenoming snakes and uniform case definitions are needed to improve comparability of different reports of neurotoxic envenoming.
4. There are many interesting acute and delayed neurotoxic manifestations other than neuromuscular weakness, and these may reveal valuable information that may lead to a better understanding of other neurological diseases.
5. The evidence for antivenom and AChEIs in treatment of neurotoxic envenoming is not strong, and large randomized trials are urgently needed.

**Five Key Papers in the Field**

1. Prasarnpun S, Walsh J, Awad SS, Harris JB (2005) Envenoming bites by kraits: the biological basis of treatment-resistant neuromuscular paralysis. Brain 128: 2987–2996.
2. Lee C, Chen D, Katz RL (1977) Characteristics of nondepolarizing neuromuscular block: (I) post-junctional block by alpha-bungarotoxin. Can Anaesth Soc J 24: 212–219.
3. Kularatne SA (2002) Common krait (*Bungarus caeruleus*) bite in Anuradhapura, Sri Lanka: a prospective clinical study, 1996–98. Postgrad Med J 78: 276–280.
4. Lalloo DG, Trevett AJ, Korinhona A, Nwokolo N, Laurenson IF, et al. (1995) Snake bites by the Papuan taipan (*Oxyuranus scutellatus canni*): paralysis, hemostatic and electrocardiographic abnormalities, and effects of antivenom. Am J Trop Med Hyg 52: 525–531.
5. Watt G, Theakston RD, Hayes CG, Yambao ML, Sangalang R, et al. (1986) Positive response to edrophonium in patients with neurotoxic envenoming by cobras (*Naja naja philippinensis*). A placebo-controlled study. N Engl J Med 315: 1444–1448.
